# Characterisation of canine CD34+/CD45 diminished cells by colony-forming unit assay and transcriptome analysis

**DOI:** 10.3389/fvets.2022.936623

**Published:** 2022-09-12

**Authors:** Taro Ayabe, Masaharu Hisasue, Yoko Yamada, Suguru Nitta, Kaoruko Kikuchi, Sakurako Neo, Yuki Matsumoto, Ryo Horie, Kosuke Kawamoto

**Affiliations:** ^1^Laboratory of Small Animal Internal Medicine, School of Veterinary Medicine, Azabu University, Sagamihara, Japan; ^2^Research and Development Section, Anicom Specialty Medical Institute Inc., Yokohama, Japan; ^3^Laboratory of Clinical Diagnosis, School of Veterinary Medicine, Azabu University, Sagamihara, Japan

**Keywords:** surface antigen, stem cell, dog, CD34, bone marrow

## Abstract

Haematopoietic stem and progenitor cells (HSPCs) are used for transplantation to reconstruct the haematopoietic pathways in humans receiving severe chemotherapy. However, the characteristics of canine HSPCs, such as specific surface antigens and gene expression profiles, are still unclear. This study aimed to characterise the haematopoietic ability and gene expression profiles of canine bone marrow HSPCs in healthy dogs. In this study, the CD34 positive (CD34+) cells were defined as classical HSPCs, CD34+/CD45 diminished (CD45^dim^) cells as more enriched HSPCs, and whole viable cells as controls. Haematopoietic abilities and gene expression profiles were evaluated using a colony-forming unit assay and RNA-sequencing analysis. Canine CD34+/CD45^dim^ cells exhibited a significantly higher haematopoietic colony formation ability and expressed more similarity in the gene expression profiles to human and mouse HSPCs than those of the other cell fractions. Furthermore, the canine CD34+/CD45^dim^ cells expressed candidate cell surface antigens necessary to define the canine haematopoietic hierarchy roadmap. These results indicate that the canine CD34+/CD45^dim^ cells express the HSPC characteristics more than the other cell fractions, thereby suggesting that these cells have the potential to be used for studying haematopoietic stem cells in dogs.

## Introduction

Haematopoietic stem and progenitor cells (HSPCs), present in the bone marrow, are multipotent cells with the ability to differentiate into mature blood cells. The haematopoietic stem cells (HSCs) have the ability to self-renew and reconstruct the haematopoietic system post-transplantation, whereas the haematopoietic progenitor cells (HPCs) have no detectable ability for self-renewal. In the classical haematopoietic hierarchy roadmap, HSCs are classified into two populations, namely, long-term (LT) HSCs and short-term (ST) HSCs, while HPCs are classified into five populations, namely, multipotent progenitors (MPPs), common lymphoid progenitors (CLPs), common myeloid progenitors (CMPs), megakaryocyte/erythrocyte progenitors (MEPs), and granulocyte/macrophage progenitors (GMPs) (1, 2). In human medicine, these HSPCs are used for haematopoietic stem cell transplantation (HSCT), and hence, they are administered to patients with haematopoietic tumour diseases after high-dose chemotherapy (3).

In veterinary medicine, autologous HSCT using peripheral blood stem cells (PBSCs) has been performed successfully in cases of canine B-cell and T-cell lymphoma; therefore, it may be considered as a new potential treatment option for canine haematopoietic tumours (4, 5). However, due to the low HSPC population in the bone marrow, it is difficult to obtain the number of HSPCs necessary for successful transplantation; hence, HSPC transplantation is uncommon in small animal practise. In human medicine, the isolation of HSPCs using cell surface antigens is critical for a successful PBSC transplantation since a high number of HSPCs contributes to an increase in the engraftment ratio post-HSCT (6). Previously, the canine CD34-positive (CD34+) cells were considered to be a definitive marker of HSCs; however, a study has revealed that the CD34+ cell population isolated from canine peripheral blood mostly consists of CD34+/CD45-high (CD45^hi^) cells and rarely the presence of CD34+/CD45-low (CD45^lo^) cells (7). Furthermore, 98% of CD34+/CD45^hi^ cells exhibited CD21-positive, thereby suggesting the presence of differentiated and maturated B-cells. Therefore, the characterisation of other cell surface antigens for the identification of canine HSPCs is necessary to develop successful HSCT in these animals.

Even though human and mouse studies have extensively reported about the cell surface protein and gene expression markers of HSPC populations, there are only a few reports on similar analysis in dogs (7–10). In general, human CD34+ cells and CD34-positive/CD45-diminished (CD34+/CD45^dim^) cells are counted to evaluate the number of HSPCs prior to transplantation. In fact, the cell surface antigens of HSPCs that are centred on CD34+ cells, such as Lineage-negative (Lin-)/CD34+/CD38– and CD34+/CD45^dim^, have generally been used in human medicine (11–14). In veterinary medicine, the CD34 antigen has been considered as a marker for HSCs, and hence, it has been used in HSCT; however, although most of the CD34+ cells were reported to be composed of differentiated B-cells, bone marrow and PBSC transplants using CD34+/CD45^dim^ have not yet been performed in dogs and cats with spontaneous haematopoietic tumors. Therefore, using CD34+/CD45^dim^ cells or hematopoietic Lin-/CD34+ cells is necessary to accurately count the canine HSPCs.

Incidentally, HSPCs have been widely studied in haematopoietic research in humans, mice, and zebrafish, and they are required as a positive control while studying haematopoietic differentiation, haematopoietic insufficiency diseases, and haematopoietic tumors. Additionally, there are several reports of canine HSPC surface antigens other than CD34, including KIT proto-oncogene receptor tyrosine kinase (KIT; CD117), FMS-like tyrosine kinase 3 (Flt-3), major histocompatibility complex (MHC) class II, and wheat germ agglutinin (WGA) (9, 15, 16); however, the functions and gene expression profiles that characterise each cell fraction are unclear with respect to veterinary medicine. Therefore, it is important to characterise the other HSPC surface antigens for the successful detection of these cells in dogs.

In this study, we analysed the haematopoietic colony forming ability and the transcriptome of canine CD34+/CD45^dim^ cells to identify potential markers for canine HSPCs.

## Materials and methods

### Animals

Clinically healthy four Beagle dogs (mean age, 5.75 ± 0.5 years) with clean medical histories were used for this study. They were kept at a small animal breeding facility in the Azabu University Veterinary Clinical Centre. All animal experiments were conducted according to the laboratory dynamic experimental guidelines of Azabu University (No. 190927-2). The dogs were not administered any drugs from 30 days prior to the beginning of the experiments.

### Preparation of canine bone marrow-derived mononuclear cells (BM-MNCs)

The dogs were sedated with medetomidine hydrochloride (0.04 mg/kg IV), butorphanol tartrate (0.02 mg/kg IV), and buprenorphine hydrochloride (0.02 mg/kg IV). Thereafter, bone marrow specimens were collected from the ilium and humerus/femur using a bone marrow aspiration needle attached to a syringe containing 1 ml heparin. The collected specimens were quickly diluted with Iscove's modified Dulbecco's medium (IMDM; Gibco, MA, USA). Subsequently, the diluted specimens were separated by centrifugation with 50 mL Leucosep (Greiner Bio-One, Frickenhausen, Germany) at 1,000 × *g* for 10 min, and the supernatant containing the BM-MNCs was transferred to another centrifuge tube, diluted with phosphate-buffered saline (PBS; Gibco), and further separated by centrifugation at 250 × *g* for 10 min. Finally, the supernatant was removed, the cell sediment was washed twice with PBS, and stored in a cell cryopreservation solution using HSC-Banker GMP grade (Zenogen Pharma, Fukushima, Japan), according to the manufacturer's protocol. The cryopreserved cells were used for further experiments.

### Flow cytometry analysis of cell surface antigen and cell sorting of canine BM-MNCs

To isolate the HSPCs, each cell fraction was assessed according to the clinical guidelines for CD34+ cell quantification in mobilised PBSCs using the sequential gating strategy of the International Society of Hematotherapy and Graft Engineering (ISHAGE) (11, 17). Our analysis was based upon the protocol described in a previous study with some modifications (18). Non-specific binding of antibodies was blocked using the Fc receptor binding inhibitor polyclonal antibody (Thermo Fisher Scientific, diluted 1:10). Phycoerythrin-conjugated mouse anti-canine CD34 (clone 1H6, BD Bioscience, diluted 1:20) and fluorescein-isothiocyanate-conjugated rat anti-canine CD45 (clone YKIX716.13, Bio-Rad, Hercules, CA, diluted 1:20) monoclonal antibodies were used for flow cytometry analysis. Additionally, phycoerythrin-conjugated mouse immunoglobulin G1 (IgG1) kappa isotype control (clone P3.6.2.8.1, Thermo Fisher Scientific, diluted 1:20) and fluorescein-isothiocyanate-conjugated rat IgG2b kappa isotype control (clone eB149/10H5, Thermo Fisher Scientific, diluted 1:20) were used as the isotype controls during the flow cytometry process. The 7-aminoactinomycin D (7-AAD; BD Bioscience, diluted 1:20) was used as the viability dye. Fluorescence-activated single cell sorting (FACS) melody (BD Bioscience, San Jose, CA, USA) was used for CD34 and CD45-based cell sorting of the canine BM-MNCs, while FlowJo (Tree Star, Ashland, OR) was used for data analysis. The HSPCs were defined as CD34+ or CD34+/CD45^dim^ cells, and they were sorted for RNA extraction and colony-forming unit assay.

### Colony-forming unit assay (CFU-A)

Haematopoietic differential ability of the sorted cells was evaluated using CFU-A. Approximately 1,000–10,000 canine BM-MNCs were sorted into three fractions, namely, whole viable cells, CD34+ cells, and CD34+/CD45^dim^ cells, according to be said cell sorting strategy. The CFU-A was performed using MethoCult H4435 enriched medium (MethoCult; Stem Cell Technologies, Vancouver, BC, Canada), which is composed of a semi-solid matrix containing supplements necessary for differentiating into mature blood cells. Each cell fraction was added to MethoCult, mixed by vigorous vortexing, and incubated at 37°C and 5% CO_2_. After 7–14 days, haematopoietic colonies, such as the burst-forming unit erythroid (BFU-E), colony-forming unit erythroid (CFU-E), colony-forming unit granulocyte (CFU-G), colony-forming unit macrophage (CFU-M), and colony-forming unit granulocyte-macrophage (CFU-GM), were observed with a phase-contrast microscope. The proportion of colony formation was calculated using the following formula: proportion of colony formation (%) = (total colony count / count of seeding cells) ×100. Statistical significance (^*^p < 0.05, ^***^p < 0.001) was assessed by one-way analysis of variance (ANOVA) using EZR (version 1.54; Jichi Medical University, Saitama, Japan) (19).

### Total RNA extraction

All four canine BM-MNC samples were subjected to RNA-sequencing (RNA-seq) analysis. The 1,000–10,000 canine BM-MNCs that had been sorted using a previously described cell sorting strategy were subsequently treated with TRIzol LS Reagent (Thermo Fisher Scientific, Waltham, MA, USA), and the total RNA was extracted using Direct-zol RNA Miniprep Plus kit (Zymo Research, Irvine, CA, USA). The RNA integrity number (RIN) was evaluated using Agilent 2100 Bioanalyzer system (Agilent Technologies, Santa Clara, CA, USA), and samples with RIN ≥ 7 were subjected to further gene expression analysis.

### Gene expression profiling by RNA-seq

A complementary DNA (cDNA) library was constructed from 250 pg of total RNA for each sample using the SMART-Seq v4 PLUS kit (TaKaRa Bio, Shiga, Japan). The cDNA library was sequenced on an Illumina NextSeq 500 (Illumina, San Diego, USA) using the NextSeq 500/550 High Output Kit v2.0 (Illumina), according to the manufacturer's protocol (Supplementary Table 1). The read length was 150 bp single-end reads. Low-quality sequenced reads were trimmed using Trimmomatic (Version 0.39) with the following parameter settings: HEADCROP: 3, CROP: 145, LEADING: 30, TRAILING: 30, SLIDEWINDOW: 10:30, MINLEN: 50, and AVGQUAL: 31. Finally, the read length was a range of 50–145 bp single-end reads. The quality of the sequenced reads was assessed using FastQC (version 0.11.9). Moreover, transcripts per million (TPM) were used for RNA quantification as well as subsequent statistical analysis. The reference cDNA datasets were CanFam3.1-based annotations deposited in the Ensembl genome browser (release 104). Gene expression was quantified using Kallisto/Sleuth pipeline. Incidentally, Kallisto (v0.46.1) is a pseudoalignment-based method used to quantify RNA abundance at the transcriptional level (20). The TPM was calculated using the quant option implemented in Kallisto. Thereafter, the downstream differential gene expression analysis was analysed by Sleuth (v0.30.0) using the TPM data (21). All statistical tests were corrected by the Benjamini–Hochberg method, and the statistical significance level was set as false discovery rate (FDR) <0.1 in the Wald test. Gene ontology (GO) analysis, a key bioinformatics tool for integrating the representation of genes and gene product attributes across all species, and Kyoto Encyclopaedia Encyclopaedia of Genes and Genomes (KEGG) pathway analysis were performed using the g:GOSt (version e104_eg51_p15_3922dba), which is a web server for performing functional enrichment analyses (22).

## Results

### Characterisation of CD34+ and CD34+/CD45^dim^ cells among canine BM-MNCs

**Figure 1:**
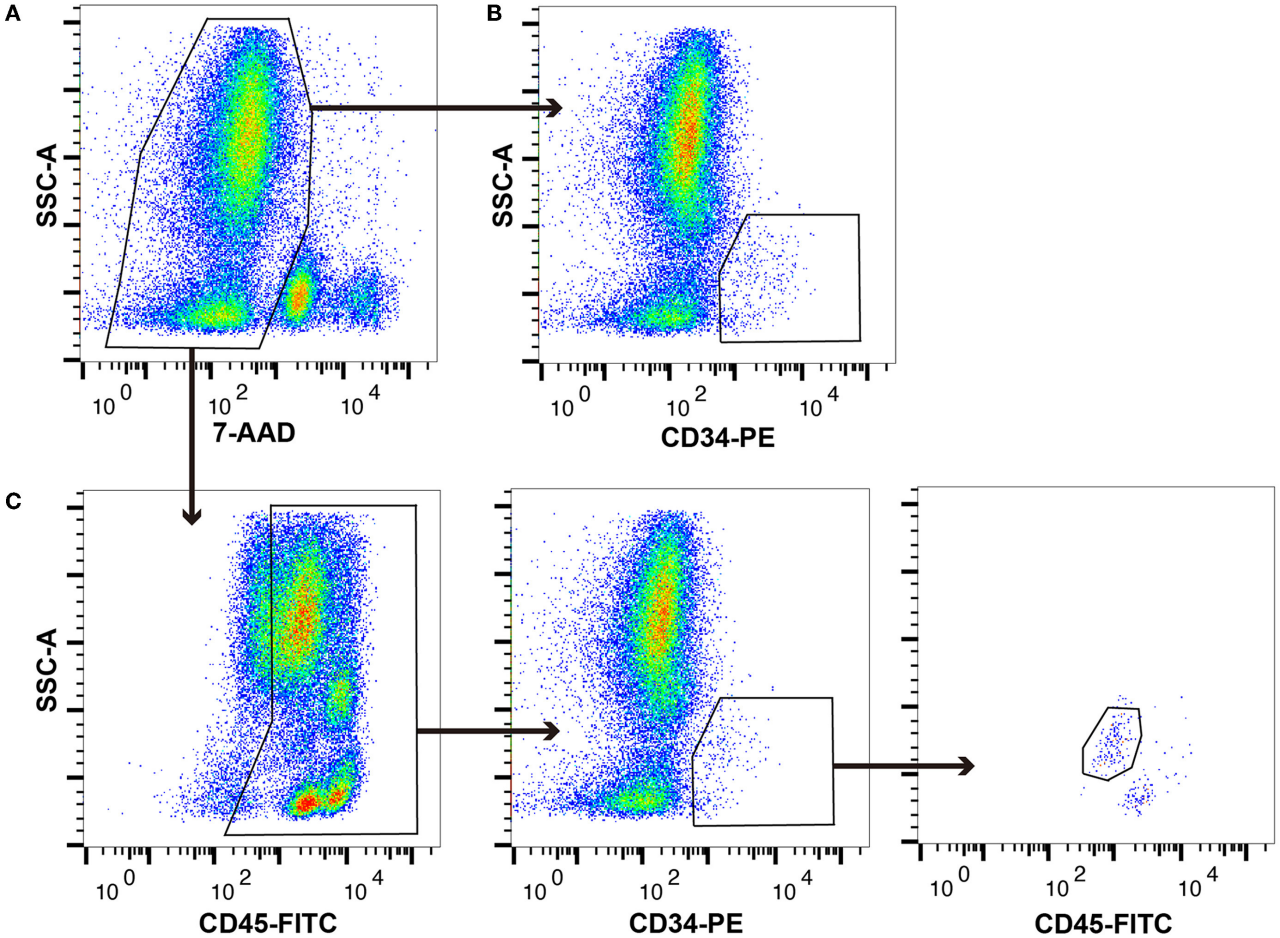
Typical plot profiles of canine haematopoietic stem and progenitor cells (HSPCs). Canine bone marrow-derived mononuclear cells (cBM-MNCs) were stained with 7-aminoactinomycin D (7-AAD), which is a viability dye, phycoerythrin (PE)-labelled anti-canine CD34 monoclonal antibody (mAb), and fluorescein isothiocyanate (FITC)-labelled anti-canine CD45 mAb. **(A)** Whole viable cells, **(B)** CD34+ cells, and **(C)** CD34+/CD45 diminished (CD45dim) cells as analysed by flow cytometry.

In this study, canine HSPCs were collected using a method similar to the ISHAGE sequential gating strategy. Typical flow cytometry plots of the canine BM-MNCs are shown in Figure 1. The proportions of whole viable cells, CD34+ cells, and CD34+/CD45^dim^ cells are 49.42 ± 3.67%, 0.36 ± 0.06%, and 0.16 ± 0.03%, respectively, among 100,000 canine BM-MNCs.

### Haematopoietic colony forming ability among canine HSPC phenotypes

**Figure 2:**
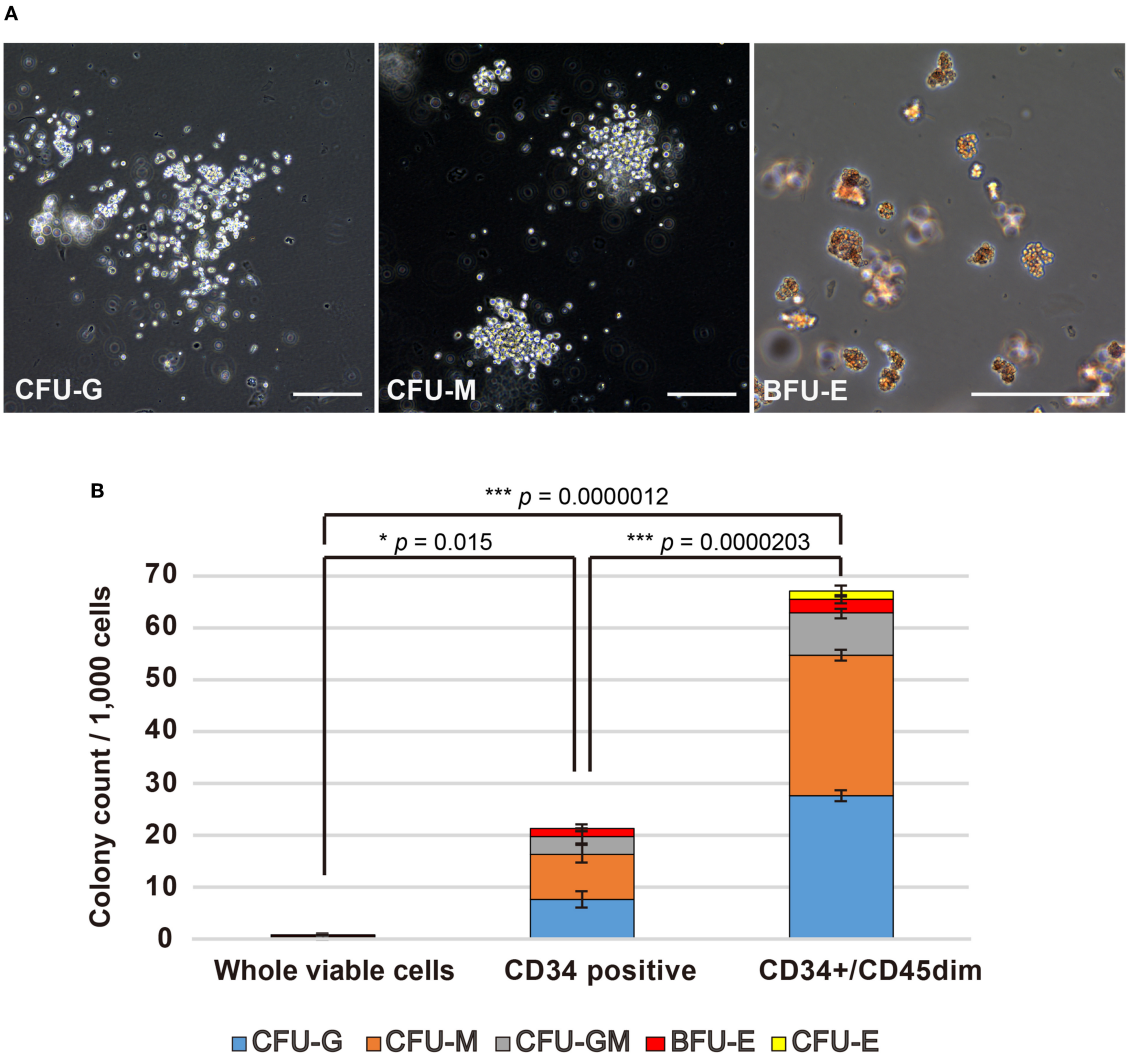
Haematopoietic colony formation by canine haematopoietic stem and progenitor cells (HSPCs). **(A)** Microscopic observation of canine haematopoietic colonies (Scale bars: 200 μm). **(B)** Haematopoietic colonies for every 1,000 cells, as counted for whole viable cells, CD34+ cells, and CD34+/CD45diminished (CD45^dim^) cells. Experiments were performed four times independently. Results are presented as mean ± standard error of the mean (SEM). Statistical significance of total colony counts was assessed by one-way analysis of variance (ANOVA). ^*^p < 0.05, ^***^p < 0.001.

To evaluate the haematopoietic multipotency of the canine HSPC phenotypes arising due to different cell surface antigens, we examined their colony forming abilities using CFU-A. In fact, each cell fraction collected during cell sorting was evaluated for its colony-forming ability. All cell fractions showed blood cell colony formation (Figure 2A). For every 1,000 canine BM-MNCs, the number of colonies formed by whole viable cells, CD34+ cells, and CD34+/CD45^dim^ cells are 0.6 ± 0.05, 19.5 ± 6.0, and 67 ± 1.85, respectively (Figure 2B). Moreover, the ability of the CD34+/CD45^dim^ cells to differentiate into haematopoietic colonies is significantly higher as compared to that of the other cell fractions. Incidentally, the CD34+/CD45^bright^/side scatter low (SSC^low^) cells were unable to form haematopoietic colonies (data not shown). Notably, there was no significant difference in the individual haematopoietic colony construction abilities of the CD34+ and CD34+/CD45^dim^ cells.

### RNA-seq analysis of canine HSPC phenotypes

**Figure 3:**
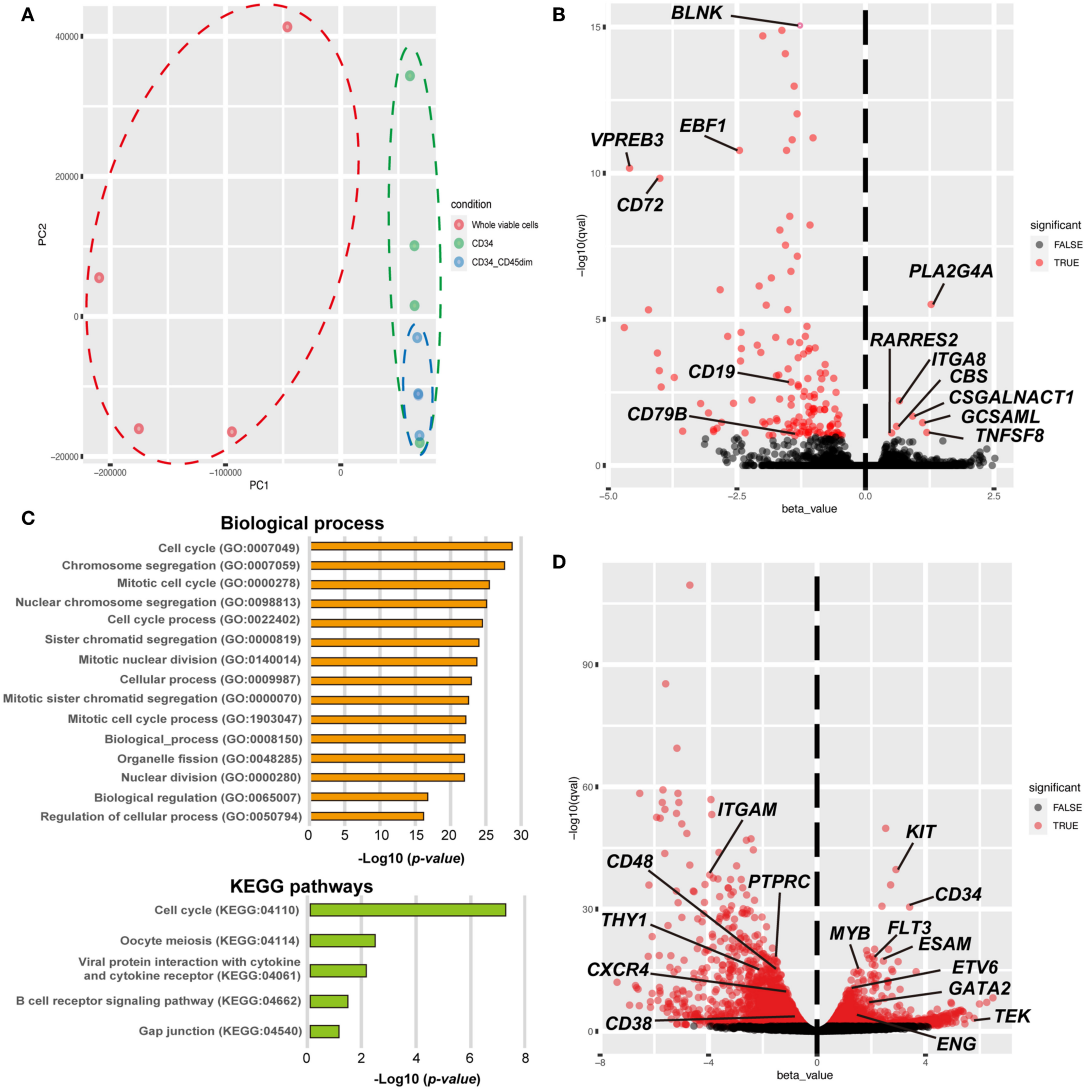
Global gene expression profiling of canine haematopoietic stem and progenitor cells (HSPCs). **(A)** Principal component analysis of the global gene expression of canine HSPCs. Gene expression is demonstrated by dots and circles of red (whole viable cells), green (CD34+ cells), and blue [CD34+/CD45 diminished (CD45dim) cells]. **(B)** The volcano plot shows the results of differentially expressed gene (DEG) analysis between CD34+/CD45dim cells and CD34+ cells. Statistical significance was set as false discovery rate (FDR) <0.1. **(C)** Gene ontology (GO) and Kyoto Encyclopaedia Gene and Genomes (KEGG)-based pathway enrichment analysis of the downregulated genes of CD34+/CD45dim cells. The candidate terms of biological processes are illustrated with adjusted *p*-values < −Log10_16_ (top). The candidate terms revealed by KEGG pathway analysis are demonstrated (bottom). **(D)** The volcano plot shows the results of DEG analysis between CD34+/CD45dim cells and whole viable cells. Statistical significance was set as FDR <0.1.

To investigate the similarities and differences between the CD34+ and CD34+/CD45^dim^ cells, we performed whole transcriptome analysis using RNA-seq. Principal component analysis demonstrates a clear separation of the whole viable cells (blue dots) from the canine HSPCs, including the CD34+ cells (red dots) and CD34+/CD45^dim^ cells (green dots) in PC1 (Figure 3A). Furthermore, the CD34+/CD45^dim^ cells are convergent, as compared to the CD34+ cells in PC2. The contribution ratio of PC1 is 93.96 % and that of PC2 is 3.46 %. Additionally, we detected 148 differentially expressed genes (DEGs) with FDR <0.1 in the canine CD34+/CD45^dim^ cells, as compared to the corresponding gene expressions in the CD34+ cells; among these 148 DEGs, 7 are upregulated and 141 are downregulated (Figure 3B). The genes that are upregulated in CD34+/CD45^dim^ cells include the ones encoding phospholipase A2 group IVA (*PLA2G4A*), integrin subunit alpha 8 (*ITGA8*), chondroitin sulfate N-acetylgalactosaminyltransferase 1 (*CSGALNACT1*), germinal centre associated signalling and motility like protein (*GCSAML*), cystathionine beta-synthase (*CBS*), TNF superfamily member 8 (*TNFSF8*), and retinoic acid receptor responder 2 (*RARRES2*). Moreover, the GO analysis of the 131 downregulated DEGs in the CD34+/CD45^dim^ cells revealed that the majority of the GO terms are associated with cell cycle and cell division, and the KEGG pathway analysis revealed that the downregulated DEGs are significantly enriched in B cell receptor signalling pathways (adjusted *p* value = 1.699 ×10^−2^) (Figure 3C). On the contrary, there are 2,286 upregulated DEGs and 2,295 downregulated DEGs in the CD34+/CD45^dim^ cells, as compared to the corresponding gene expressions in the whole viable cells (Figure 3D). The upregulated DEGs associated with the major surface antigens of HSPCs are *KIT* proto-oncogene (*KIT*; CD117), *CD34*, FMS-like tyrosine kinase 3 (*FLT3*; CD135), endothelial cell adhesion molecule (*ESAM*), *TEK Receptor Tyrosine Kinase* (*TEK*; Tie2), and endoglin (*ENG*; CD105), while the downregulated DEGs are integrin alpha M (*ITGAM*; CD11b), protein tyrosine phosphatase receptor type C (*PTPRC*; CD45RA), *CD48, Thy-1 cell surface antigen* (*THY1*; CD90), CXC chemokine receptor type 4 (*CXCR4*), and *CD38*. Additionally, *MYB Proto-Oncogene* (*MYB*), ETS variant transcription factor 6 (*ETV6*), GATA binding factor 2 (*GATA2*), homeobox A5 (*HOXA5*), *HOXA7, HOXA9*, and *HOXA10*, which are highly expressed in HSCs, are upregulated in the CD34+/CD45^dim^ cells.

## Discussion

This study demonstrates that the canine CD34+/CD45^dim^ cells have a high haematopoietic colony forming ability and a high degree of HSPC-associated gene expression pattern, indicating their potential to function as canine HSPCs. Generally, HSPCs are collected directly from the bone marrow by aspiration, or they are mobilised into peripheral blood and collected by apheresis (1). Moreover, low population cells, like these HSPCs, are generally analysed after enrichment or by single-cell analysis using flow cytometric cell sorting (23, 24). In this study, the canine CD34+ and CD34+/CD45^dim^ cells had a very low population in the bone marrow; therefore, they were subjected to cell sorting enrichment prior to any experiments (Figure 1). With respect to veterinary medicine, Faldyna et al. reported the presence of 1%−3% CD34+ cells (15), and Tsumagari et al. reported the presence of 0.30 ± 0.07% CD34+/CD45^dim^ cells in the adult canine bone marrow (7). Interestingly, the population of CD34+/CD45^dim^ cells (0.16 ± 0.03%) recorded in this study is lower than that reported in the previous studies. However, the reason for this striking difference is unknown. We believe that this low population of CD34+/CD45^dim^ cells in this study might be due to the exclusion of doublets, dead cells, and surrounding fraction cells.

In this study, the canine CD34+/CD45^dim^ cells exhibited a high cell differentiation ability, i.e., their colony forming ability was significantly greater than that of CD34+ cells and whole viable cells (Figure 2B). Interestingly, a study related to human medicine reports that the CD45 antigen expression is lower in the immature cells of the early differentiation stages than that in the mature lymphocytes and monocytes (17). Furthermore, the expression level of CD45 antigen is low to moderate, even in the CD34+ HSPCs, and the endothelial cells, the other cell types of CD34+ cells, exhibit negative expression of CD45 antigens. Therefore, the combination of CD34 and CD45 makes it possible to distinctly select the HSPCs. Moreover, we did not observe any differences between the CD34+/CD45^dim^ and CD34+ cell fractions with respect to the BFU-E, CFU-E, CFU-G, CFU-M, and CFU-GM types of haematopoietic colony formations. This suggests that CD34+/CD45^dim^ cells did not inhibit the differentiation of any specific cell lineage of the canine HSPCs. On the contrary, CFU-A of canine CD34+/CD45^bright^/SSC^low^ cells demonstrated their inability to form haematopoietic colonies. A previous study had revealed that the canine CD34+ cell fraction mostly consisted of B-cells and exhibited non-specific binding to anti-CD34 antibodies (7). This suggests that the CD34+/CD45^bright^/SSC^low^ cells are non-HSPCs, including other haematopoietic cell lineages.

Based on transcriptome analysis, we showed that the canine CD34+/CD45^dim^ cells have a gene expression profile similar to that of the HSPCs. Moreover, the principal component of the canine CD34+/CD45^dim^ cells gene expression was concentrated with similar components compared to whole viable cells and CD34+ cells. Additionally, a DEG analysis between the canine CD34+/CD45^dim^ cells and the CD34+ cells revealed that four out of the seven upregulated DEGs in the CD34+/CD45^dim^ cells, (*PLA2G4A, CSGALNACT1, CBS*, and *RARRES2)* were reportedly associated with HSPCs (25–30), and the remaining three (*ITGA8, GCSAML*, and *TNFSF8*) with haematopoietic lineage cells (31–37). The *PLA2G4A* encodes a key enzyme that catalyses the hydrolysis of membrane phospholipids to release arachidonic acid (38). The *CSGALNACT1* encodes a key enzyme in chondroitin sulphate biosynthesis (27), and in *CSGALNACT1*-knockout mice, there is a reported disruption of HSC maintenance and early T-cell development (28). Previous studies have demonstrated a higher expression of these two genes in HSPCs, as compared to that in myelocytes or lymphocytes (26, 27), and in this study, these two gene expressions were significantly increased in the canine CD34+/CD45^dim^ cells than in the CD34+ cells. The CBS is a key enzyme for hydrogen sulphide (H_2_S) production from L-cysteine, and CBS deficiency induces a disruption of erythropoiesis by interfering with the expression of haem-biosynthetic enzymes and haem-transporters (29). The *RARRES2*, which encodes a protein named chemerin, is secreted from Lin-/Sca-1+/c-kit+/CD34- LT-HSC in mice, and *RARRES2* neutralisation blocks osteoclastogenesis of HSCs (30). These reports demonstrate that the abovementioned genes play an important role in HSPC functions and haematopoiesis, and the upregulation of these genes characterises canine CD34+/CD45^dim^ cells as HSPCs.

Furthermore, we detected 141 downregulated DEGs in the canine CD34+/CD45^dim^ cells as compared to the corresponding gene expressions in the CD34+ cells, and a GO analysis of 131 of these DEGs revealed numerous GO annotations associated with cell cycle and cell division. This suggests that the canine CD34+/CD45^dim^ cells have a longer quiescent phase of the cell cycle than the CD34+ cells (Figure 3C). Under normal conditions, the bone marrow is in a hypoxic environment, and majority of the HSPCs are maintained in the quiescent G_0_ phase by hypoxia-inducible factor 1-alpha (HIF-1α) (39). Additionally, the HSPCs are tightly regulated with respect to quiescence, proliferation, and differentiation in the bone marrow microenvironment or niche (40). Another study has reported that HSPCs are maintained by CXC chemokine ligand (CXCL)12-abundant reticular (CAR) cells, and they form bone marrow microenvironments known as haematopoietic stem cell niches (41). Therefore, it is possible that the canine bone marrow HSCs contain quiescent phase cells. This supports the presence of a high number of quiescent phase cells among the canine CD34+/CD45^dim^ cells.

The KEGG pathway enrichment analysis revealed that the downregulated DEGs are associated with the B cell receptor signalling pathway (Figure 3C). A previous report revealed that canine CD34+ cell fraction contained B-cells that exhibited non-specific binding to anti-CD34 antibodies (7). Therefore, in this study, the canine CD34+ cell fraction also included other kinds of HSPCs. We have demonstrated that the major surface antigen genes associated with HSPCs are differentially expressed in CD34+/CD45^dim^ cells than in the whole viable cells (Figure 3D). In humans and in mice, KIT, CD34, FLT3, Tie2, CD105, CD45RA, and CD90 are generally used as surface antigens to define the haematopoietic hierarchy roadmap (13, 42–45). Moreover, CD38, which is expressed in many cell types, including plasma cells, is generally used as a negative HSC marker in humans (12, 46). The ESAM is a novel, human primitive HSC marker that plays an important role in definitive haematopoiesis development (47). These antigen expressions are common to humans and mice; therefore, they can be considered as candidates for determining the haematopoietic hierarchy roadmap in dogs, as well. Additionally, *MYB, ETV6, GATA2, HOXA5, HOXA7, HOXA9*, and *HOXA10*, which are reportedly HSPC markers (48–51), were upregulated in the canine CD34+/CD45^dim^ cells, thereby supporting the characterisation of canine CD34+/CD45^dim^ cells as HSPCs.

According to the observations of this study, CD34+/CD45^dim^ seems to have the potential to function as a surface antigen marker for the identification of canine HSPCs. Canine HSPC surface antigens have not been elucidated clearly, and the definition of the haematopoietic hierarchy roadmap has been incomplete in veterinary medicine. McSweeney et al. have reported that CD34 is expressed in the canine bone marrow cells, and CD34+ cells have a haematopoietic colony forming ability, thereby suggesting their probable characterisation as HSPCs (52). Furthermore, cryopreserved CD34+ cells can retain their colony formation ability (53). Kim et al. demonstrated that the comparative study regarding the safety of PBSC collection methods by apheresis in healthy dogs using CD34+/CD45^dim^ cells (18). Incidentally, the molecular-level characteristics of CD34+/CD45^dim^ cells were not assessed in that study; however, it is necessary to analyse whether the CD34+/CD45^dim^ cells have the cell differentiation potential and haematopoietic stem cell characteristics suitable for induction in transplantation therapy. In this study, we have successfully demonstrated that canine CD34+/CD45^dim^ cells exhibit more HSPC characteristics than the CD34+ cells.

In the clinical practise of human medicine, HSPCs have been extensively used as a tumour treatment option. In fact, HSCT is performed by autologous and allogeneic methods, both of which generally use PBSCs collected by apheresis. Autologous transplantation has the advantage of avoiding graft-vs.-host disease (GvHD), and it has a reduced relapse risk (RR) over allogeneic transplantation (54). Even though allogeneic transplantation has an increased GvHD risk in some recipients, it has been widely used to attack and eradicate malignant cells, in which the donor immune cells are capable of eradicating chemotherapy-resistant tumour cells by the graft-versus-tumour (GVT) effect (55). Meanwhile, pluripotent stem cells, such as embryonic stem cells (ESCs) and induced pluripotent stem cells (iPSCs), can differentiate into various types of cells and tissues, and many studies have reported haematopoietic differentiation methods *via* HSPCs using pluripotent stem cells in humans and mice (56–59). Therefore, it is expected that allogeneic HSCTs will be performed increasingly in the future, and the analysis of immunogenicity and cell surface markers will be important for determining the safety of transplantation in canines and humans.

There are a few limitations in this study. First, we did not confirm the haematopoietic reconstruction ability *in vivo* by transplantation of CD34+/CD45^dim^ cells into immune-deficient mice. Second, the CD34+/CD45^dim^ cells are not sufficient to exclusively confirm the presence of HSCs; therefore, the location of the HSPCs among the canine CD34+/CD45^dim^ cells is still unclear with respect to the classical haematopoietic hierarchy Roadmap. Additional Markers Need to be Determined for This Purpose.

## Conclusion

These results indicate that the canine CD34+/CD45^dim^ cells conformed to the characteristics of HSPCs based upon the ISHAGE sequential gating strategy, especially in terms of haematopoietic colony forming ability and gene expression profile. Moreover, it contributes to the determination of candidate surface antigens that define the canine haematopoietic hierarchy roadmap. We believe that these results will lead to long-term haematopoietic reconstructions in HSCT and efficient haematopoietic differentiation from pluripotent stem cells in dogs. However, further research is needed for the exclusive identification of canine HSPCs in the future.

## Data availability statement

The datasets presented in this study can be found in online repositories. The names of the repository/repositories and accession number(s) can be found below: Submission: DRA014248, Run: DRR378823-DRR378834. BioProject: PRJDB13629 (PSUB017583), BioSample: SAMD00493090-SAMD00493092 (SSUB021958), SAMD00493093-SAMD00493095 (SSUB021989), SAMD00493096-SAMD00493098 (SSUB021990), and SAMD00493099-SAMD00493101 (SSUB021991).

## Ethics statement

The animal study was reviewed and approved by Animal experiment Committee of Azanu University.

## Author contributions

TA, MH, and KKa contributed to conception and design of the study. YM and RH organised the database. TA, SNi, YY, SNe, and KKi performed the experiment. TA and MH wrote the first draught of the manuscript. All authors contributed to manuscript revision, read, and approved the submitted version.

## Funding

This study was supported by the Grant and Academic Consulting Fee of Laboratory of Small Animal Internal Medicine, Azabu University, and Research Foundation of Anicom Specialty Medical Institute Inc. (Japan).

## Conflict of interest

Authors TA, YM, and KKa were employed by Anicom Specialty Medical Institute Inc. The remaining authors declare that the research was conducted in the absence of any commercial or financial relationships that could be construed as a potential conflict of interest.

## Publisher's note

All claims expressed in this article are solely those of the authors and do not necessarily represent those of their affiliated organizations, or those of the publisher, the editors and the reviewers. Any product that may be evaluated in this article, or claim that may be made by its manufacturer, is not guaranteed or endorsed by the publisher.

## Abbreviations

HSPC: haematopoietic stem and progenitor cell
HSC: haematopoietic stem cell
HPC: haematopoietic progenitor cell
HSCT: haematopoietic stem cell transplantation
LT-HSC: long-term HSC
ST-HSC: short-term HSC
MPP: multipotent progenitor
CLP: common lymphoid progenitor
CMP: common myeloid progenitor
MEP: megakaryocyte/erythrocyte progenitor
GMP: granulocyte/macrophage progenitor
BM-MNC: bone marrow-derived mononuclear cells
Flt-3: FMS-like tyrosine kinase 3
MHC: major histocompatibility complex
CFU-A: colony forming unit assay
BFU-E: burst forming unit-erythroid
CFU-E: colony forming unit-erythroid
CFU-G: colony forming unit granulocyte
CFU-M: colony forming unit macrophage
CFU-GM: colony forming unit granulocyte-macrophage
TPM: transcripts per million
PLA2G4A: phospholipase A2 group IVA
ITGA8: integrin subunit alpha 8
CSGALNACT1: chondroitin sulphate N-acetylgalactosaminyltransferase 1
GCSAML: germinal centre associated signalling and motility like
CBS: cystathionine beta-synthase
TNFSF8: TNF superfamily member 8
RARRES2: retinoic acid receptor responder 2
ITGAM: integrin alpha M
PTPRC: protein tyrosine phosphatase receptor type C
CXCR4: CXC chemokine receptor type 4
ETV6: ETS variant transcription factor 6
GATA2: GATA binding factor 2
HOXA5: homeobox A5
ESAM: endothelial cell adhesion molecule
ENG: endoglin
DEG: differentially expressed gene
GvHD: graft-vs.-host disease
GvT: graft-vs.-tumor.
