# Supplementary material for: Characterisation of canine CD34+/CD45 diminished cells by colony-forming unit assay and transcriptome analysis
Source: Front Vet Sci. 2022 Sep 12;9:936623. doi: 10.3389/fvets.2022.936623 (PMC9510753; doi:10.3389/fvets.2022.936623)
Supplement: Supplementary file 1 [file Data_Sheet_1.PDF]

Supplementary Table 1. DDBJ Sequence Read Archive (DRA) accession number and details of data contents. Accession number: DRA014248. HSPCs: haematopoietic stem and progenitor cells, CD45dim: CD45 diminished.

DRA Run Accession	Sample Name	Cell type
**DRR378823**	Canine_HSPCs_1-1	whole viable cells
**DRR378824**	Canine_HSPCs_1-2	CD34+ cells
**DRR378825**	Canine_HSPCs_1-3	CD34+/CD45dim cells
**DRR378826**	Canine_HSPCs_2-1	whole viable cells
**DRR378827**	Canine_HSPCs_2-2	CD34+ cells
**DRR378828**	Canine_HSPCs_2-3	CD34+/CD45dim cells
**DRR378829**	Canine_HSPCs_3-1	whole viable cells
**DRR378830**	Canine_HSPCs_3-2	CD34+ cells
**DRR378831**	Canine_HSPCs_3-3	CD34+/CD45dim cells
**DRR378832**	Canine_HSPCs_4-1	whole viable cells
**DRR378833**	Canine_HSPCs_4-2	CD34+ cells
**DRR378834**	Canine_HSPCs_4-3	CD34+/CD45dim cells
